# Strong and Selective Inhibitory Effects of the Biflavonoid Selamariscina A against CYP2C8 and CYP2C9 Enzyme Activities in Human Liver Microsomes

**DOI:** 10.3390/pharmaceutics12040343

**Published:** 2020-04-10

**Authors:** So-Young Park, Phi-Hung Nguyen, Gahyun Kim, Su-Nyeong Jang, Ga-Hyun Lee, Nguyen Minh Phuc, Zhexue Wu, Kwang-Hyeon Liu

**Affiliations:** 1 BK21 Plus KNU Multi-Omics based Creative Drug Research Team, College of Pharmacy and Research Institute of Pharmaceutical Sciences, Kyungpook National University, Daegu 41566, Korea; soyoung561021@gmail.com (S.-Y.P.); lgh2710@gmail.com (G.-H.L.); 2 College of Pharmacy and Research Institute of Pharmaceutical Sciences, Kyungpook National University, Daegu 41566, Korea; hyunlove9137@naver.com (G.K.); phucnguyen0606@gmail.com (N.M.P.); wuzhexue527@gmail.com (Z.W.); 3 Institute of Natural Products Chemistry, Vietnam Academy of Science and Technology, 18-Hoang Quoc Viet, Cau Giay, Hanoi 100000, Vietnam; nguyenphihung1002@gmail.com; 4 Vietnam Hightech of Medicinal and Pharmaceutical JSC, Group 11 Quang Minh town, Hanoi 100000, Vietnam

**Keywords:** biflavonoid, cytochrome P450, drug interactions, selamariscina A, uridine 5′-diphosphoglucuronosyl transferase

## Abstract

Like flavonoids, biflavonoids, dimeric flavonoids, and polyphenolic plant secondary metabolites have antioxidant, antibacterial, antiviral, anti-inflammatory, and anti-cancer properties. However, there is limited data on their effects on cytochrome P450 (P450) and uridine 5′-diphosphoglucuronosyl transferase (UGT) enzyme activities. In this study we evaluate the inhibitory potential of five biflavonoids against nine P450 activities (P450s1A2, 2A6, 2B6, 2C8, 2C9, 2C19, 2D6, 2E1, and 3A) in human liver microsomes (HLMs) using cocktail incubation and liquid chromatography-tandem mass spectrometry (LC–MS/MS). The most strongly inhibited P450 activity was CYP2C8-mediated amodiaquine *N*-dealkylation with IC50 ranges of 0.019~0.123 μM. In addition, the biflavonoids—selamariscina A, amentoflavone, robustaflavone, cupressuflavone, and taiwaniaflavone—noncompetitively inhibited CYP2C8 activity with respective *K*_i_ values of 0.018, 0.083, 0.084, 0.103, and 0.142 μM. As selamariscina A showed the strongest effects, we then evaluated it against six UGT isoforms, where it showed weaker inhibition (UGTs1A1, 1A3, 1A4, 1A6, 1A9, and 2B7, IC50 > 1.7 μM). Returning to the P450 activities, selamariscina A inhibited CYP2C9-mediated diclofenac hydroxylation and tolbutamide hydroxylation with respective *K*_i_ values of 0.032 and 0.065 μM in a competitive and noncompetitive manner. However, it only weakly inhibited CYP1A2, CYP2B6, and CYP3A with respective *K*_i_ values of 3.1, 7.9, and 4.5 μM. We conclude that selamariscina A has selective and strong inhibitory effects on the CYP2C8 and CYP2C9 isoforms. This information might be useful in predicting herb-drug interaction potential between biflavonoids and co-administered drugs mainly metabolized by CYP2C8 and CYP2C9. In addition, selamariscina A might be used as a strong CYP2C8 and CYP2C9 inhibitor in P450 reaction-phenotyping studies to identify drug-metabolizing enzymes responsible for the metabolism of new chemicals.

## 1. Introduction

Flavonoids are polyphenolic secondary metabolites that are common in the plant kingdom and are ingested by humans in their food [1]. Flavonoids are grouped into various classes based on structure. These classes are: anthocyanidins, chalcones, flavanones, flavones, flavonols, isoflavonoids, and biflavonoids [2]. Many pharmacological benefits have been ascribed to flavonoids, including antioxidant, anti-inflammatory, anti-cancer, antiviral, and hepatoprotective effects [3,4].

Having flavonoids in your diet may reduce the risk of atherosclerosis, cardiovascular disease, diabetes mellitus, osteoporosis, and certain cancers [4,5]. Because of flavonoids’ benefits and wide distribution, their intake has risen steadily in recent years in the West and Asia. Daily intake of flavonoids has been estimated at 100 mg/day in the Asian population because of the high consumption of soy products [6,7]. On the other hand, daily intake of flavonoids has been estimated to be in the range of 20–50 mg/day in Western populations [8]. Further intake of flavonoids through dietary supplements and plant extracts with prescribed drugs is common. The vast body of literature describes the significant interactions between flavonoid herbs and therapeutic drugs.

Several flavonoids are substrates for cytochrome P450 (P450) and uridine 5′-diphosphoglucuronosyl transferase (UGT) enzymes [2], suggesting that flavonoids could inhibit the activities of these enzymes. A number of studies have demonstrated that flavonoids are potent inhibitors of CYP1A2, CYP3A, and UGT1A1 in vitro [5,8]. For example, the flavone tangeretin competitively inhibits the activity of CYP1A2 with a *K*i value as low as 68 nM in human liver microsomes (HLMs) [9]. It also inhibits UGT1A1-mediated estradiol glucuronidation with an IC50 value of 1 μM [10]. The flavonols quercetin and kaempferol inhibit the metabolism of nifedipine and felodipine by CYP3A4 in HLMs at concentrations larger than 10 μM. [11]. Animal studies show that oral quercetin increases the bioavailability of oral doxorubicin [12]. These results can be attributed to the reduced first-pass metabolism of doxorubicin due to quercetin-induced inhibition of CYP3A and/or enhanced doxorubicin absorption in the gastrointestinal tract via quercetin-induced inhibition of P-glycoprotein (P-gp). Surya Sandeep et al. (2014) reported that naringenin significantly increases the bioavailability of orally administered felodipine, a P-glycoprotein and CYP3A4 substrate drug, in rats, through the inhibition of intestinal P-gp and CYP3A4 [13]. Alnaqeeb et al. (2019) reported that quercetin and guava leaf extracts in combination with warfarin exert a greater increase on warfarin’s *C*_max_ and International Normalized Ratio values than when used alone, indicating the inhibition of CYP2C8, 2C9 and 3A4, major warfarin-metabolizing enzymes [14]. Biflavonoids, formed by the covalent bond between two monoflavonoids, are a subclass of flavonoid [15]. They are secondary metabolites, but are limited to several species in plants such as *Ginkgo biloba*, *Selaginella* species, *Hypericum perforatum*, and *Garcinia kola* [16]. Befitting their status as flavonoids, they have anti-cancer, anti-microbial, antiviral, and anti-inflammatory properties [16]. In contrast to the extensive studies on drug interaction with flavonoids, data on the inhibitory effects of biflavonoids on P450 and UGT enzymes are rare, though biflavonoids are taken in the form of dietary supplements (e.g., *Ginkgo biloba* extract [17]). The inhibitory potential of amentoflavone, the major biflavonoid in *Cupressus funebris*, against P450 and UGT enzymes was only recently reported [18,19].

**Figure 1:**
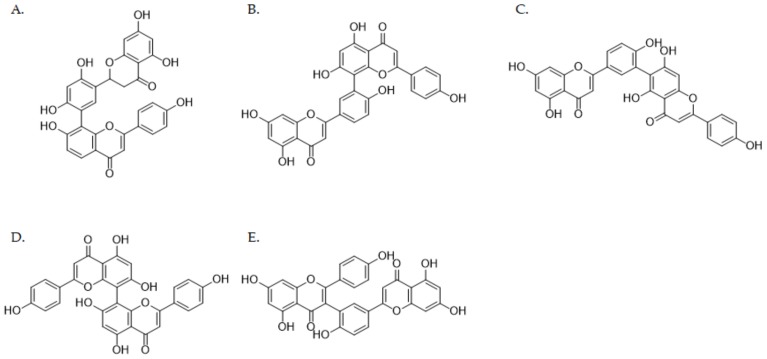
Chemical structures of biflavonoids from *Selaginella tamariscina*: selamariscina A (**A**), amentoflavone (**B**), robustaflavone (**C**), cupressuflavone (**D**) and taiwaniaflavone (**E**).

In this study, we evaluate the inhibitory effects of five biflavonoids—selamariscina A, amentoflavone, robustaflavone, cupressuflavone, and taiwaniaflavone (Figure 1)—on nine P450 enzymes using HLMs. We further investigate the ability of selamariscina A, which most strongly inhibited CYP2C8 and CYP2C9 activities, to inhibit six UGT isoforms. Furthermore, the inhibition mechanism and kinetic parameters (*K*_i_) were determined for selamariscina A and compared with those of montelukast, a well-known selective CYP2C8 inhibitor [20].

## 2. Materials and Methods

### 2.1. Chemicals and Reagents

We purchased acetaminophen, alamethicin, amodiaquine, bupropion, chenodeoxycholic acid, chlorzoxazone, dextromethorphan, estrone glucuronide, glucose-6-phosphate (G6P), glucose-6-phosphate dehydrogenase (G6PDH), hydroxybupropion, magnesium chloride (MgCl2), N-acetylserotonin, β-nicotinamide adenine dinucleotide phosphate (NADP+), N-desethylamodiaquine, omeprazole, phenacetin, trifluoperazine, trimipramine, and uridine 5′-diphosphoglucuronic acid (UDPGA) from Sigma-Aldrich (St. Louis, MO, USA). 4-Hydroxydiclofenac, 4-hydroxytolbutamide, 5-hydroxyrosiglitazone, coumarin, diclofenac, midazolam, montelukast, mycophenolic acid, rosiglitazone, and tolbutamide came from Toronto Research Chemicals (Toronto, ON, Canada). We obtained 1′-hydroxymidazolam from Cayman Chemical (Ann Arbor, MI, USA), while 7-ethyl-10-hydroxycomptothecine (SN-38) was provided by Santa Cruz Biotechnology (Dallas, TX, USA). All solvents were LC–MS grade (Fisher Scientific, Pittsburgh, PA, USA). All the other reagents were of analytical or LC–MS grade and are commercially available. We purchased the pooled human liver microsomes (XTreme 200) from XenoTech (Lenexa, Kansas City, KS, USA). In this study, we used selamariscina A, amentoflavone, robustaflavone, cupressuflavone, and taiwaniaflavone identified from Selaginella tamariscina (Beauv.), which were collected at Yen Tu Mountain, Uong Bi town, Quang Nihn province, Vietnam. The information regarding the identification of their chemical structures was described in our previously published paper [21,22].

We isolated selamariscina A, amentoflavone, robustaflavone, cupressuflavone, and taiwaniaflavone from Selaginella tamariscina (Beauv.), which were collected at Yen Tu Mountain, Uong Bi town, Quang Nihn province, Vietnam. The five compounds were purified and examined by HPLC to get 95% purity. Their chemical structures were identified by analyzing their NMR data, which were in good agreement with those published in a previous report [21,22].

### 2.2. Inhibitory Effect of Five Biflavonoids against Human Cytochrome P450 Activity

The inhibitory potential of the five biflavonoids on the metabolism of nine P450 probe substrates was evaluated using previously developed methods with minor modifications [23,24]. Biflavonoids were dissolved in methanol. The final concentration of methanol in the incubation mixture was 1.0% (*v*/*v*). We used these P450 probe substrates: phenacetin for CYP1A2, coumarin for CYP2A6, bupropion for CYP2B6, amodiaquine for CYP2C8, diclofenac for CYP2C9, omeprazole for CYP2C19, dextromethorphan for CYP2D6, chlorzoxazone for CYP2E1 and midazolam for CYP3A (Table 1). The incubation mixtures containing pooled human liver microsomes (HLMs, XTreme 200, XenoTech), P450 probe substrates, and inhibitor (0~20 µM) were pre-incubated at 37 °C for 5 min. The concentration range of the inhibitor varied (0, 0.002, 0.005, 0.02, 0.05, and 0.2 µM for CYP2C8; 0, 0.02, 0.05, 0.2, 0.5, and 2 µM for CYP2C9; 0, 0.5, 2, 5, 10, and 20 µM for other P450 isoforms). After pre-incubation, a reduced nicotinamide adenine dinucleotide phosphate (NADPH) generation system containing 1 unit/ml G6PDH, 1.3 mM β- nicotinamide adenine dinucleotide phosphate (β- NADP^+^), 3.3 mM MgCl_2_, and 3.3 mM G6P was added to initiate a reaction, and further incubated for 10 min at 37 °C. The reaction was stopped by adding 50 μL of ice-cold acetonitrile containing 7 nM trimipramine (internal standard, IS). After centrifugation at 18,000 g (5 min, 4 °C), aliquots of supernatants were analyzed by LC–MS/MS (Shimadzu LCMS 8060 system, Shimadzu, Kyoto, Japan). All microsomal incubations were conducted in triplicate.

### 2.3. Kinetic Characterization of Five Biflavonoids on CYP2C8 in Human Liver Microsomes

To determine the inhibition mechanism and constants (Ki values) of the five biflavonoids against CYP2C8 activity, different concentrations of biflavonoids (0, 0.002, 0.005, 0.02, 0.05, and 0.2 μM for selamariscina A; 0, 0.05, 0.02, 0.05, 0.2 and 0.5 μM for the other four biflavonoids) were added to reaction mixtures containing different concentrations of amodiaquine (0.1, 0.4 and 1 μM). The other conditions were the same as in the cytochrome P450 inhibition study.

### 2.4. Kinetic Characterization of Selamariscina A on Five P450 Enzymes in Human Liver Microsomes

We used HLMs to determine the mechanisms and constants (Ki values) for selamariscina A inhibition of CYP1A2, CYP2B6, CYP2C8, CYP2C9 and CYP3A. The selamariscina A (0~50 μM) was added into the reaction mixtures, each of which contained concentrations of phenacetin (20, 50, and 100 μM), bupropion (20, 50, and 100 μM), amodiaquine (0.1, 0.4, and 1 μM), rosiglitazone (2, 5, and 10 μM), diclofenac (1, 4, and 10 μM), tolbutamide (50, 100, and 200 μM), and midazolam (0.5, 2, and 5 μM). The substrates were used at concentrations approximately near to their respective Km values [25,26,27]. The concentration range of selamariscina A varied (0, 0.002, 0.005, 0.02, 0.05, and 0.2 μM for CYP2C8; 0, 0.05, 0.02, 0.05, 0.2, and 0.5 μM for CYP2C9; 0, 0.2, 0.5, 2, 5, and 20 µM for CYP3A; 0, 0.5, 2, 5, 20, and 50 μM for CYP1A2 and CYP2B6). The other conditions were the same as in the cytochrome P450 inhibition study.

### 2.5. Time-Dependent Inhibition Assay

The time-dependent inhibition of selamariscina A against CYP2C8 and CYP2C9 enzymes was evaluated using an IC50 shift method. Selamariscina A was pre-incubated at six concentrations (0, 0.002, 0.005, 0.02, 0.05, and 0.2 μM) with HLMs in the presence of an NDAPH generation system for 30 min at 37 °C. The reaction was initiated by adding 1 μM amodiaquine or 10 μM diclofenac and further incubated for 10 min. Incubation was terminated by adding 50 μL of ice-cold acetonitrile containing 7 nM trimipramine. After centrifugation, aliquots of supernatants were analyzed by LC–MS/MS.

### 2.6. Inhibitory Effect of Selamariscina A against Human UGT Activity

The ability of selamariscina A to inhibit the metabolism of six UGT enzyme probe substrates was evaluated using previously developed methods with minor modifications [28]. The microsomal incubation was performed by dividing the non-interactive substrate cocktail sets (set A included SN-38 for UGT1A1, CDCA for UGT1A3 and TFP for UGT1A4 while set B included N-SER for UGT1A6, MPA for UGT1A9 and NX for UGT2B7) (Table 2). In brief, HLMs (0.25 mg/mL) were activated by incubation in the presence of alamethicin (25 µg/mL) for 15 min on ice. After the addition of UGT probe substrates and inhibitor (0, 0.5, 2, 5, 20 and 50 μM), the incubation mixtures were pre-incubated at 37 °C for 5 min. After pre-incubation, 5 mM UDPGA was added to initiate a reaction, and further incubated for 60 min at 37 °C. The reaction was stopped by adding 50 μL of ice-cold acetonitrile containing 250 nM estrone glucuronide (IS). After centrifugation at 18,000 g (5 min, 4 °C), aliquots of supernatants were analyzed by LC–MS/MS. All microsomal incubations were conducted in triplicate.

### 2.7. LC–MS/MS Analysis

All metabolites and the IS were separated on a Kinetex XB-C18 column (100 × 2.10 mm, 2.6 μm, 100 Å; Phenomenex, Torrance, CA, USA) and analyzed using a Shimadzu LCMS 8060 triple-quadrupole mass spectrometer coupled with a Nexera X2 ultra high-performance liquid chromatography system (Shimadzu, Kyoto, Japan) equipped with an electrospray ionization interface. The mobile phase consisted of 0.1% formic acid in water (A) and 0.1% formic acid in acetonitrile (B). The elution condition was set as 8% B (0–0.5 min), 8%→60% B (0.5–5 min), 60% B (5–6 min), 60%→8% B (6–6.1 min) and 8% B (6.1–9 min) for the analysis of metabolites of P450 probe substrates and set as 0%→40% B (0–1 min), 40%→50% B (1–5 min), 50%→0% B (5–5.1 min), and 0% B (5.1–8 min) for the analysis of metabolites of UGT probe substrates. The flow rate was 0.2 mL/min. Electrospray ionization was performed in positive-ion mode at 4000 V or in negative-ion mode at −3500 V. The optimum operating conditions were determined as follows: vaporizer temperature, 300 °C; capillary temperature, 350 °C; collision gas (argon) pressure, 1.5 mTorr. Quantitation was conducted in selected reaction monitoring (SRM) modes with the precursor-to-product ion transition for each metabolite (Table 1 and Table 2).

### 2.8. Data Analysis

We analyzed the data with Shimadzu LabSolution LC–MS software. The IC50 values were calculated by WinNonlin software (Pharsight, Mountain View, CA, USA). The type of inhibition and the apparent kinetic parameters for inhibitory activity (*K*i) were determined by following several criteria: visual inspection of Dixon plots, Lineweaver–Burk double reciprocal plots, and secondary plots of Lineweaver–Burk plots versus biflavonoid concentrations, the size of the sum of squares of the residuals, Akaike Information Criteria values, the S.E. and 95% confidence interval of the parameter estimates from the nonlinear regression analysis [29] using the WinNonlin software. The models tested included competitive, competitive partial, noncompetitive, noncompetitive partial, uncompetitive, uncompetitive partial, and mixed-type inhibition.

## 3. Results and Discussion

### 3.1. Inhibition of Cytochrome P450 Enzymes Activities by Five Biflaovnoids

The inhibitory potential of the five biflavonoids against cytochrome P450 enzyme activity was evaluated using HLMs (Table 3). We found selamariscina A, amentoflavone, robustaflavone, cupressuflavone, and taiwaniaflavone strongly inhibit CYP2C8 activity with respective IC50 values of 0.019, 0.084, 0.083, 0.083, and 0.12 μM. They also show strong inhibition on CYP2C9 activity with IC50 values of 0.047, 0.15, 0.15, 0.21, and 0.20 μM. Their inhibition of the other seven P450 isoforms was much lower (IC50 ≥ 1.2 µM) than on CYP2C8 and CYP2C9 (IC50 ≤ 0.21 µM). The IC50 value of the inhibition of diclofenac hydroxylase activity by amentoflavone that we found (0.15 µM) is 4.3 times higher than the 0.035 µM reported by von Moltke et al. (2004) [19]. The reason could be differences in incubation conditions, such as CYP2C19 probe substrate and concentrations (diclofenac 10 µM versus S-mephenytoin 25 µM) [30]. The inhibitory potential (IC50 = 1.3 µM) of amentoflavone on CYP3A was similar to the previously reported value (IC50 = 4.3 µM) [19].

As the five flavonoids strongly inhibited microsomal CYP2C8 activity, we sought to clarify the mechanism of inhibition. The Lineweaver–Burk plots, Dixon plots and secondary reciprocal plots indicated that selamariscina A, amentoflavone, robustaflavone, cupressuflavone, and taiwaniaflavone noncompetitively inhibited CYP2C8-mediated amodiaquine N-deethylation activity with Ki values of 0.018, 0.083, 0.084, 0.103, and 0.142 μM, respectively (Table 4), which are lower than those of the well-known CYP2C8 inhibitors zafirlukast (0.39 μM) [31] and quercetin (4.72 μM) [32].

**Figure 2:**
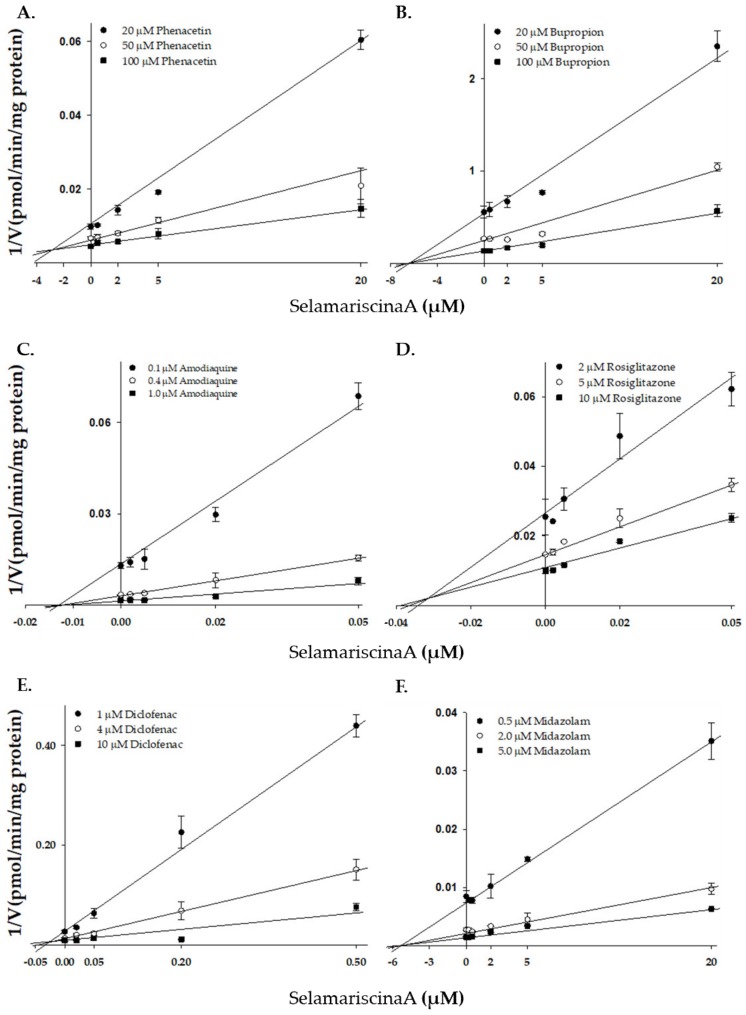
Representative Dixon plots obtained from a kinetic study of CYP1A2-catalyzed phenacetin *O*-deethylation (**A**), CYP2B6-catalyzed bupropion hydroxylation (**B**), CYP2C8-catalyzed amodiaquine N-deethylation (**C**), CYP2C8-catalyzed rosiglitazone 5-hydroxylation (**D**), CYP2C9-catalyzed diclofenac 4-hydroxylation (**E**), and CYP3A-catalyzed midazolam 1′-hydroxylation (**F**) in the presence of different concentrations of selamariscina A in pooled human liver microsomes (XTreme 200, XenoTech). Each data point shown represent the mean ± standard error in triplicate for the samples.

Of the five biflavonoids, selamariscina A most strongly inhibited CYP2C8-mediated amodiaquine N-deethylation activity with a Ki value of 0.018 µM, which is similar to the IC50 value of the known strong CYP2C8 inhibitor montelukast (0.020 μM) [31]. Further, its inhibitory potential against CYP2C8 was much stronger than other known CYP2C8 inhibitors axitinib (Ki = 0.17 µM [33]), clotrimazole (IC50 = 0.78 µM [31]), felodipine (IC50 = 1.20 µM [31]), nilotinib (Ki = 0.10 µM [33]), and quercetin (Ki = 1.56 µM [34]). We further evaluated the inhibition mechanism of selamariscina A, which showed the strongest CYP2C8 inhibition, for the other P450 enzymes. The inhibitory potential (Ki) of selamariscina A against P450 enzyme activities was in the order: CYP2C8 > CYP2C9 > CYP1A2 > CYP3A > CYP2B6 (Table 5, Figure 2 and Figure S1). To determine whether inhibition by selamariscina A was substrate-specific, we evaluated its inhibitory effects on CYP2C8-mediated rosiglitazone 5-hydroxylation. We found that it showed strong inhibition with a Ki value of 0.010 μM in a substrate-independent manner. Selamariscina A also inhibited CYP2C9-mediated diclofenac and tolbutamide hydroxylation with Ki values of 0.032 and 0.065 μM, respectively, in a substrate-independent manner. Its inhibitory potential against CYP2C9 was much stronger than other known CYP2C8 inhibitors sulfaphenazole (Ki = 0.12–0.7 µM [35]), fluvoxamine (Ki = 0.63–16 µM [35]), fluconazole (Ki = 0.28 µM [36]), and fluoxetine (Ki = 19–87 µM [35]). Its inhibitory potential for CYP2C8 and CYP2C9 was much stronger than other P450s.

In addition, several P450 inhibitors including azamulin, clopidogrel, methoxalene, and ticlopidine [37,38,39] have been shown to be time-dependent inhibitors of cytochrome P450. We investigated the effect of incubation time on IC50 values of selamariscina A using the CYP2C8 substrate amodiaquine and the CYP2C9 substrate diclofenac. The inhibitory potential of selamariscina A against CYP2C8-mediated amodiaquine O-deethylase activity and CYP2C9-mediated diclofenac hydroxylase activity in HLMs pre-incubated in the presence of an NADPH generation system (IC50 values of 0.031 and 0.092 μM, respectively) was a bit weaker than in untreated HLMs (IC50 values of 0.019 and 0.054 μM, respectively). This suggests that selamariscina A is not a time-dependent inhibitor (data are not shown).

### 3.2. Inhibition of UGT Enzymes Activities by Selamariscina A

The inhibitory potential of selamariscina A against uridine 5′-diphosphoglucuronosyl transferase (UGT) activity was evaluated using HLMs (Table 6). Selamariscina A inhibited UGT1A1 and UGT1A4 activity with IC50 values of 1.7 and 7.7 μM, respectively. However, its inhibition of UGT1A1 and UGT1A4 isoforms was much weaker than that of CYP2C8 (IC50 = 0.019 µM). The inhibitory potential of selamariscina A for UGT1A3, UGT1A6, UGT1A9, and UGT2B6 was negligible (IC50 > 40 µM). The IC50 value of the inhibition of UGT1A1 activity by amentoflavone found in our study (1.7 µM) is similar to its previously reported value (IC50 = 0.78 µM) [18].

### 3.3. Comparison of the Selectivity of Selamariscina A and Montelukast for CYP2C8 Inhibition

**Figure 3:**
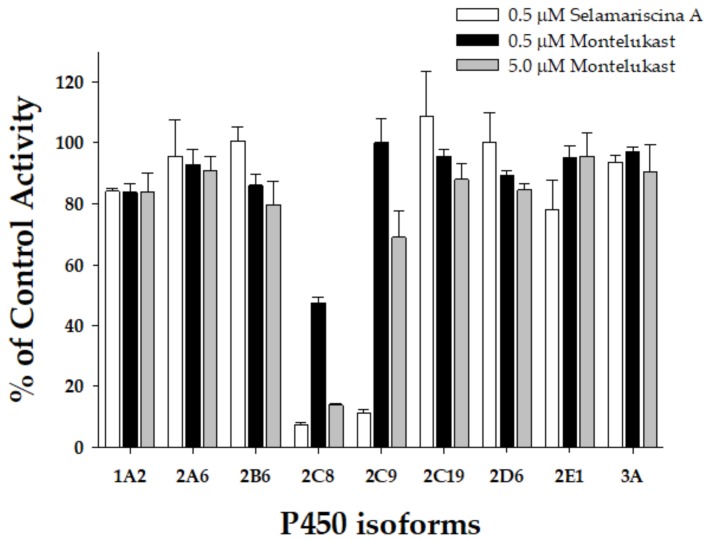
Inhibitory effects of selamariscina A (0.5 μM, ☐) and montelukast (0.5 μM, ■; 5 μM, ■) on the enzymatic activities of nine P450 isoforms in pooled human liver microsomes (0.25 mg/mL, XTreme 200, XenoTech). Phenacetin (100 μM), coumarin (5 μM), bupropion (50 μM), amodiaquine (1 μM), diclofenac (10 μM), omeprazole (20 μM), dextromethorphan (5 μM), chlorzoxazone (50 μM), and midazolam (5 μM) were used as the respective substrates of P450s 1A2, 2A6, 2B6, 2C8, 2C9, 2C19, 2D6, 2E1, and 3A. The data are means of the average ± standard error in triplicate.

Montelukast has been used to inhibit CYP2C8 in reaction-phenotyping studies [20]. We re-evaluated its inhibitory potential against the nine P450 isoforms in this study using HLMs (XTreme 200, XenoTech). Montelukast strongly inhibited CYP2C8 activity with an IC50 value of 0.52 μM, but it showed weak inhibition on the other eight P450 enzymes (IC50 > 9.73 μM) (Table 3). The IC50 value for the CYP2C8 isoform (IC50 = 0.52 μM at 0.25 mg/mL microsomal protein concentration) was similar to previously reported values (IC50 = 0.18 μM at 0.3 mg/mL microsomal protein concentration) [20]. However, montelukast showed more than 25 times weaker inhibition than selamariscina A (IC50 = 0.019 μM at 0.25 mg/mL microsomal protein concentration). At 0.5 μM selamariscina A concentration, approximately 25 times greater than the Ki value, selamariscina A was found to inhibit CYP2C8 and CYP2C9 by 92.8% and 88.6% respectively, and only slightly affected the enzyme activities of the other P450 isoforms (Figure 3). Selamariscina A at 0.5 μM concentration inhibited none of the other P450 isoform-specific activities above 21.8% in HLMs, indicating that selamariscina A could be used as a selective CYP2C8 and CYP2C9 inhibitor in P450 phenotyping studies. Montelukast at 0.5 μM concentration, a well-known selective CYP2C8 inhibitor [20], showed moderate inhibition on CYP2C8 by 52.7% in pooled HLMs. At 5 μM concentration, montelukast inhibited CYP2C8 by 86.1% in HLMs; however, it also inhibited CYP2C9 and CYP2B6 activities by 31.0% and 20.4%, respectively in pooled HLMs. Montelukast (5 μM) showed negligible inhibition on the other six P450 isoforms. Selamariscina A could be useful as a strong CYP2C8 and CYP2C9 inhibitor in P450 reaction-phenotyping studies.

### 3.4. Evaluation of Drug Interaction Potential of Selamariscina A

It was estimated that an in vivo interaction potential via the inhibition of P450 would likely occur if the ratio of inhibitor *C*_max_/*K*_i_ exceeded one and would be possible if it was between 0.1 and 1.0 [40,41]. Based on amentoflavone’s maximum concentrations (0.041 and 0.063 μM) in rat blood after a single oral dose of *Selaginella doderleinii* Hieron extracts (200 mg/kg; contents: 103.82 mg/g amentoflavone, 37.52 mg/g robustaflavone, 44.4 mg/g 2,″3′-dihydro-3′,3″-biapigenin, 53.4 mg/g 3′,3″-binaringenin, and 35.1 mg/g delicaflavone) [42] and *Selaginella doderleinii* Hieron extracts (600 mg/kg) [43], the respective values of *C*_max_/*K*_i_ were 0.49 and 0.76 from the data of pooled HLMs (*K*_i_ = 0.083 μM), indicating that amentoflavone has possible drug interaction potential with CYP2C8 substrate drugs [44]. Recently, nanotechnology-based delivery systems such as liposomes have been developed for improving oral bioavailability [42]. The values of *C*_max_ (0.22 μM) of amentoflavone after administration of liposome-based *Selaginella doderleinii* Hieron extracts (200 mg/kg) were 5.4 times higher than those of the control [42], resulting in a *C*_max_/*K*_i_ value of 2.65, indicating that amentoflavone has drug interaction potential. In the case of selamariscina A, the present study provides the first published data on its pharmacokinetics in animals and humans. Therefore, it is difficult to estimate the drug interaction potential of selamariscina A for humans. However, selamariscina A might have drug interactions with CYP2C8 substrate drugs such as cerivastatin [45], paclitaxel [46], and rosiglitazone [47] because its CYP2C8 inhibitory potential was more than 4.5 times stronger than that of amentoflavone. Therefore, in vivo studies are necessary to determine whether drug interactions between selamariscina A and CYP2C8 or CYP2C9 substrates have clinical relevance.

## 4. Conclusions

In conclusion, we report that selamariscina A is a strong CYP2C8 and CYP2C9 inhibitor. When evaluated for amodiaquine *O*-deethylation and diclofenac hydroxylation inhibitory activity against CYP2C8 and CYP2C9, as well as seven other P450s, it exhibited above 50-fold selectivity. Like montelukast and sulfaphenazole, selamariscina A could be useful as a strong CYP2C8 and CYP2C9 inhibitor in P450 phenotyping studies when HLMs are the enzyme source. Additionally, selamariscina A might cause clinically relevant pharmacokinetic drug interactions with other co-administered drugs metabolized by CYP2C8 or CYP2C9. These in vitro findings provide primary data for future in vivo animal and clinical studies on risk prediction related to the interaction of drugs with herbs.

## Figures and Tables

**Table 1:** Selected reaction monitoring (SRM) condition for the major metabolites of the nine cytochrome P450 probe substrates and internal standard (IS).

P450 Enzyme	Substrates	Concentration (μM)	Metabolites	SRM Transition (*m*/*z*)	Polarity	Collision Energy (eV)
1A2	Phenacetin	100	Acetaminophen	152 > 110	ESI^+^	25
2A6	Coumarin	5	7-Hydroxycoumarin	163 > 107	ESI^+^	17
2B6	Bupropion	50	6-Hydroxybupropion	256 > 238	ESI^+^	20
2C8	Amodiaquine	1	*N*-Desethylamodiaquine	328 > 283	ESI^+^	17
	Rosiglitazone	5	*p*-Hydroxyrosiglitazone	374 > 151	ESI^+^	17
2C9	Tolbutamide	100	4-Hydroxytolbutamide	287 > 89	ESI^+^	60
	Diclofenac	10	4-Hydroxydiclofenac	312 > 231	ESI^+^	15
2C19	Omeprazole	20	5-Hydroxyomeprazole	362 > 214	ESI^+^	10
2D6	Dextromethorphan	5	Dextrorphan	258 > 157	ESI^+^	35
2E1	Chlorzoxazone	50	6-Hydroxychlorzoxazone	184 > 120	ESI^−^	18
3A	Midazolam	5	1′-Hydroxymidazolam	342 > 203	ESI^+^	25
IS	Trimipramine	0.007		295 > 100	ESI^+^	17

ESI: Electrospray ionization (ESI) interface to generate protonated ions [M+H]^+^ or deprotonated ion [M−H]^−.^

**Table 2:** Selected reaction monitoring (SRM) condition for the major metabolites of the six uridine 5′-diphosphoglucuronosyl transferase (UGT) enzyme substrates and internal standard (IS).

UGT Enzyme	Substrates	Concentration (μM)	Metabolites	SRM Transition (*m*/*z*)	Polarity	Collision Energy (eV)
1A1	SN-38	0.5	SN-38 glucuronide	569 > 393	ESI^+^	30
1A3	Chenodeoxycholic acid (CDCA)	2	CDCA-24 glucuronide	567 > 391	ESI^−^	20
1A4	Trifluoperazine (TFP)	0.5	TFP *N*-glucuronide	584 > 408	ESI^+^	30
1A6	*N*-Acetylserotonin (*N*-SER)	1	*N*-SER glucuronide	395 > 219	ESI^+^	10
1A9	Mycophenolic acid (MPA)	0.2	MPA 7-*O*-glucuronide	495 > 319	ESI^−^	25
2B7	Naloxone (NX)	0.2	NX 3-glucuronide	504 > 310	ESI^+^	30
IS	Estrone glucuronide	0.25		445 > 269	ESI^−^	35

**Table 3:** Inhibitory effects of five biflavonoids and montelukast against nine cytochrome P450 isoforms.

P450 Enzyme	Substrate	IC_50_ (µM)					
		Selamaris-Cina A	Amento-Flavone	Robusta-Flavone	Cupressu-Flavone	Taiwania-Flavone	Montelukast
1A2	Phenacetin	7.4	4.4	4.5	5.9	6.8	>50
2A6	Coumarin	11.6	11.9	11.8	>20	10.6	>50
2B6	Bupropion	5.3	7.1	5.7	6.7	6.4	>50
2C8	Amodiaquine	0.019	0.084	0.083	0.083	0.12	0.52
2C9	Diclofenac	0.047	0.15	0.15	0.21	0.20	9.73
2C19	Omeprazole	13.3	3.4	6.4	3.0	5.0	>50
2D6	Dextromethorphan	10.6	2.6	2.2	2.7	3.2	>50
2E1	Chlorzoxazone	>20	3.3	2.9	2.3	6.0	>50
3A	Midazolam	2.7	1.3	1.2	1.5	1.2	>50

**Table 4:** *K*_i_ values for inhibition of CYP2C8-catalyzed amodiaquine *N*-deethylation in human liver microsomes by five biflavonoids.

P450 Enzyme	Substrate	Inhibitor	*K*_i_ (µM) ^a^	Mode of Inhibition
CYP2C8	Amodiaquine	Selamariscina A	0.018 ± 0.002	Noncompetitive
		Amentoflavone	0.083 ± 0.009	Noncompetitive
		Robustaflavone	0.084 ± 0.016	Noncompetitive
		Cupressuflavone	0.103 ± 0.017	Noncompetitive
		Taiwaniaflavone	0.142 ± 0.026	Noncompetitive

^a^ Values represent the average ± standard error in triplicate.

**Table 5:** *K*_i_ values for the inhibition of CYP1A2-catalyzed phenacetin *O*-deethylation, CYP2B6-catalyzed bupropion hydroxylation, CYP2C8-catalyzed amodiaquine *N*-deethylation, CYP2C8-catalyzed rosiglitazone 5-hydroxylation, CYP2C9-catalyzed diclofenac 4-hydroxylation, CYP2C9-catalyzed tolbutamide 4-hydroxylation, and CYP3A-catalyzed midazolam 1′-hydroxylation in human liver microsomes by selamariscina A.

P450 Enzyme	Substrate	*K*_i_ (µM) ^a^	Mode of Inhibition
1A2	Phenacetin	3.1 ± 0.6	Competitive
2B6	Bupropion	7.9 ± 1.1	Noncompetitive
2C8	Amodiaquine	0.018 ± 0.002	Noncompetitive
	Rosiglitazone	0.010 ± 0.003	Noncompetitive, partial
2C9	Diclofenac	0.032 ± 0.007	Competitive
	Tolbutamide	0.065 ± 0.01	Noncompetitive
3A	Midazolam	4.5 ± 0.5	Noncompetitive

^a^ Values represent the average ± standard error in triplicate.

**Table 6:** Inhibitory effects of selamariscina A against six uridine 5′-diphosphoglucuronosyl transferase (UGT) isoforms.

UGT Enzyme	Substrate	IC50 (µM) ^a^
1A1	SN-38 *	1.7 ± 0.5
1A3	Chenodeoxycholic acid	>50
1A4	Trifluoperazine	7.7 ± 1.9
1A6	*N*-Acetylserotonin	46.1 ± 11.7
1A9	Mycophenolic acid	40.4 ± 11.1
2B7	Naloxone	>50

* SN-38: 7-Ethyl-10-hydroxy camptothecin; ^a^ values represent the average ± standard error in triplicate.

## Supplementary Materials

The following are available online at https://www.mdpi.com/1999-4923/12/4/343/s1, Figure S1: Representative Lineweaver–Burk plots obtained from a kinetic study of CYP1A2-catalyzed phenacetin *O*-deethylation (A), CYP2B6-catalyzed bupropion hydroxylation (B), CYP2C8-catalyzed amodiaquine *N*-deethylation (C), CYP2C8-catalyzed rosiglitazone 5-hydroxylation (D), CYP2C9-catalyzed diclofenac 4-hydroxylation (E), and CYP3A-catalyzed midazolam 1′-hydroxylation (F) in the presence of different concentrations of selamariscina A. Each data point shown represent the mean ± standard error in triplicate samples.
