# Supplementary material for: Strong and Selective Inhibitory Effects of the Biflavonoid Selamariscina A against CYP2C8 and CYP2C9 Enzyme Activities in Human Liver Microsomes
Source: Pharmaceutics. 2020 Apr 10;12(4):343. doi: 10.3390/pharmaceutics12040343 (PMC7238120; doi:10.3390/pharmaceutics12040343)
Supplement: Supplementary file 1 [file pharmaceutics-12-00343-s001.pdf]

pharmaceutics

MDPI

Supplementary Materials: Strong and Selective Inhibitory Effects of the Biflavonoid Selamariscina A against CYP2C8 and CYP2C9 Enzyme Activities in Human Liver Microsomes

So-Young Park, Phi-Hung Nguyen, Gahyun Kim, Su-Nyeong Jang, Ga-Hyun Lee, Nguyen Minh Phuc, Zhexue Wu and Kwang-Hyeon Liu

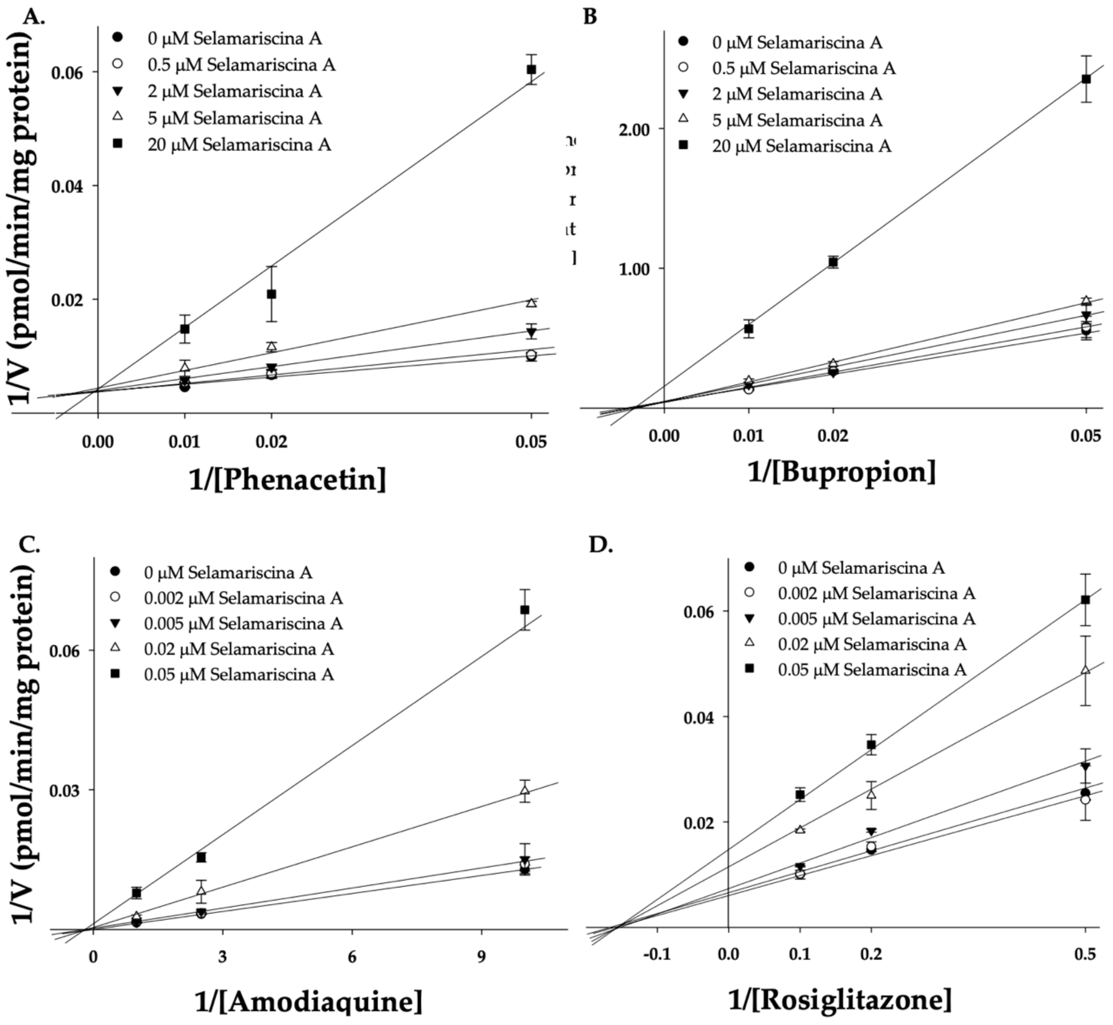

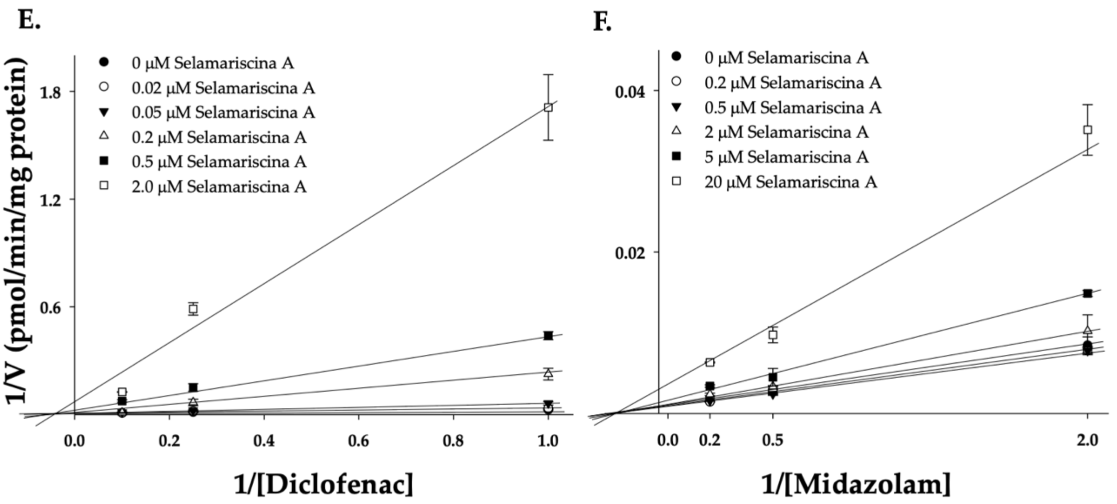

Figure S1. Representative Lineweaver-Burk plots obtained from a kinetic study of CYP1A2-catalyzed phenacetin *O*-deethylation (A), CYP2B6-catalyzed bupropion hydroxylation (B), CYP2C8-catalyzed amodiaquine *N*-deethylation (C), CYP2C8-catalyzed rosiglitazone 5-hydroxylation (D), CYP2C9-catalyzed diclofenac 4-hydroxylation (E), and CYP3A-catalyzed midazolam 1′-hydroxylation (F) in the presence of different concentrations of selamariscina A. Each data points shown represent the mean ± standard error of triplicate samples.
